# Internal thoracic artery patch repair of a saccular left main coronary artery aneurysm

**DOI:** 10.1186/s13019-019-0868-0

**Published:** 2019-02-26

**Authors:** Tomohiro Iwakura, Kouji Toguchi, Ippei Kato, Noriko Asakawa

**Affiliations:** Department of Cardiovascular Surgery, Meirikai Chuo General Hospital, 114-0001 Higashi-jyujo Kita-ku, Tokyo, Japan

**Keywords:** Coronary artery aneurysm, Left main coronary artery, Saccular aneurysm, Internal thoracic artery patch repair, Coronary bypass grafting, Stenosis

## Abstract

**Background:**

A saccular aneurysm located at the bifurcation of the left main coronary artery (LMCA) is an extremely rare condition. A major cause of left main coronary aneurysm is atherosclerosis, and common complications include thrombosis, embolism, and rupture. Despite the serious nature of this condition, the ideal operative approach to LMCA aneurysm (LMCAA) has not been established. Furthermore, little is known about resection of the saccular aneurysm and closure using a small internal thoracic artery patch.

**Case presentation:**

Here, we present the case of a 66-year-old woman who had significant stenosis in the left anterior descending artery and a saccular aneurysm at the bifurcation of the LMCAA, which was repaired using a small internal thoracic artery patch during coronary artery bypass grafting. Postoperative multislice computed tomography revealed the complete disappearance of the aneurysm and a successful repair with no luminal stenosis of the internal thoracic artery patch. In addition, the left internal thoracic artery graft was found to be patent.

**Conclusions:**

Resection of the saccular LMCA aneurysm and closure using a small internal thoracic artery patch is safe and offer excellent results.

## Background

A saccular aneurysm located at the bifurcation of the left main coronary artery (LMCA) is extremely rare, having been recorded in only 22 of 22,000 (0.1%) catheterizations by Topaz et al. [1] The anatomic location of the aneurysm and involvement of the coronary artery can complicate interventions. As a result, the ideal operative approach for an LMCA aneurysm (LMCAA) is still unclear. In this report, we present the case of successful surgical resection of a saccular LMCA aneurysm and closure using a small internal thoracic artery patch and concomitant construction of a left internal thoracic artery (LITA) graft to address significant stenosis in the left anterior descending (LAD) artery.

## Case presentation

**Fig. 1:**
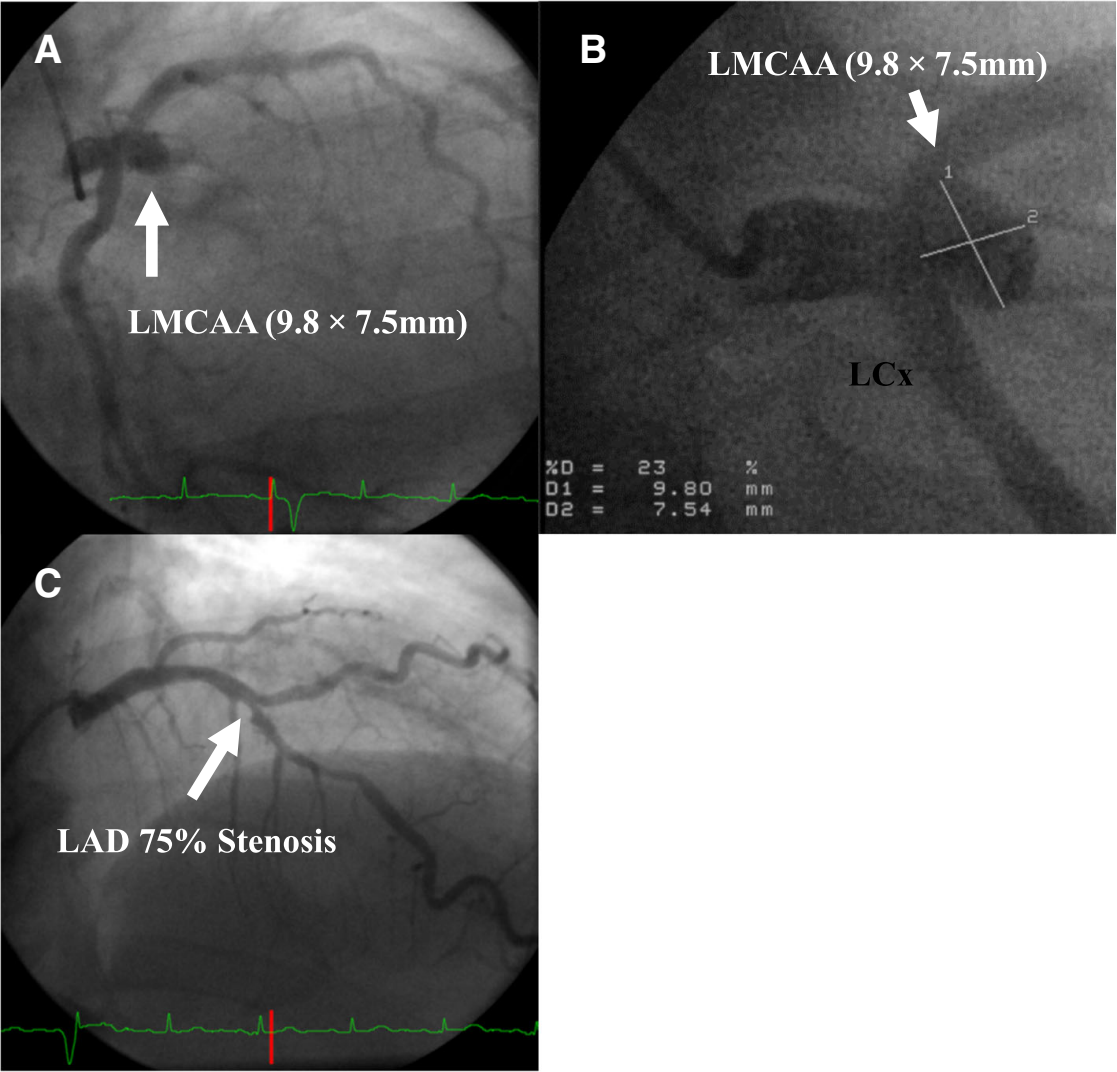
**a**, **b** Coronary angiogram showing a saccular aneurysm (arrow) at the left main coronary artery bifurcation. **c** Left anterior descending (LAD) arteries (arrow) showing 75% stenosis. LMCAA: left main coronary artery aneurysm

**Fig. 2:**
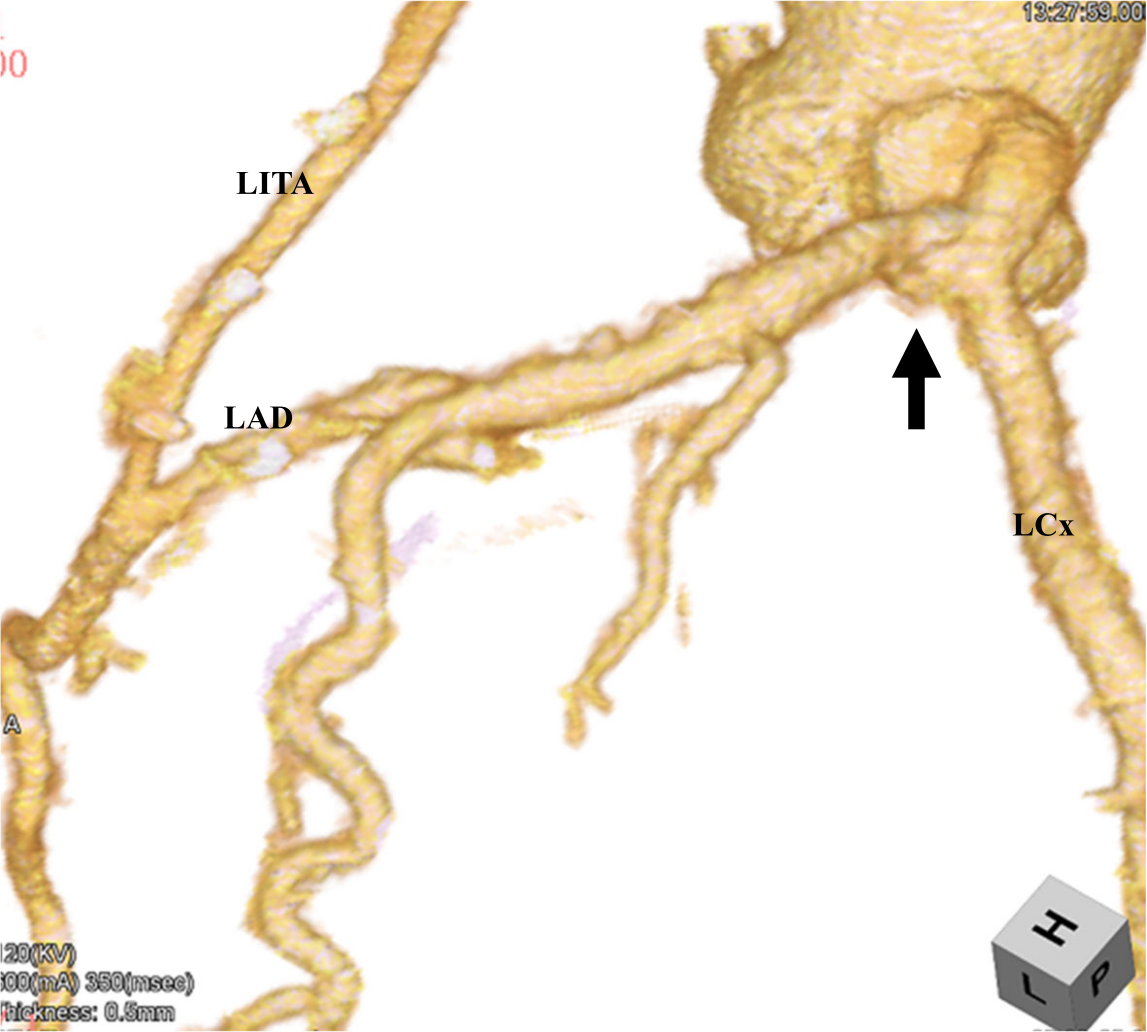
Multislice computed tomography revealing the repair of the LMCAA (arrow) with an internal thoracic artery patch and patent grafts after surgery. There was no pseudoaneurysm associated with the patch repair. LMCAA: left main coronary artery aneurysm, LAD: left anterior descending artery, LCx: left circumflex artery, LITA: left internal thoracic artery

**Fig. 3:**
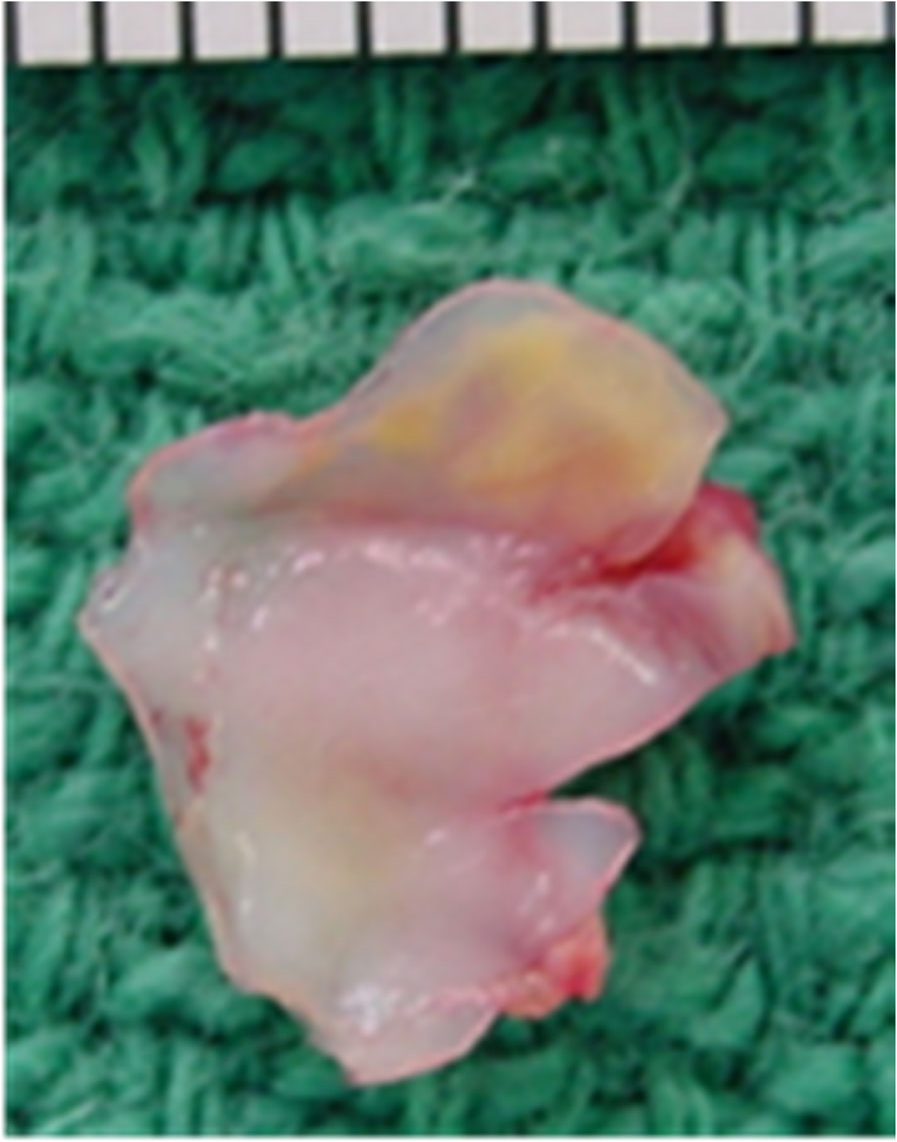
The resected saccular left main coronary aneurysm specimen showing significant atherosclerosis and partial vessel wall thinning

A 66-year-old woman presented at a hospital with chest pain. Her history included diabetes, hypertension, and hyperlipidemia. On examination, the patient had a pulse of 100 beats/minute and blood pressure of 150/80 mmHg. Her electrocardiogram, echocardiogram, and blood test results were normal. Multislice computed tomography (CT) showed a saccular LMCA aneurysm and significant stenosis in the LAD artery (Fig. 1). Coronary angiography revealed a saccular LMCA aneurysm measuring 9.8 × 7.5 mm with 75% stenosis in the proximal portion of the LAD artery. The operation was performed under general anesthesia. A median sternotomy was performed, and after a longitudinal pericardial opening was made, the heart was inspected. The LITA was removed from the inner chest wall in a skeletonized fashion using electric cautery. A distal segment of 1.5–2 cm was procured and reserved for use as a patch repair. Before aortic cannulation, the ascending aorta was dissected from the pulmonary artery. Under cardiopulmonary bypass, coronary artery bypasses of the left internal thoracic artery to the LAD artery were constructed in the beating heart. After aortic cross-clamping, the LMCA saccular aneurysm was exposed without main pulmonary artery transection. The saccular LMCA aneurysm was carefully dissected and completely excised. There was no thrombus in the lumen. Then, the LITA was longitudinally divided and trimmed to fit the incised LMCA. The small internal thoracic artery patch was sutured to the normal and firm lateral coronary arterial wall with a continuous 7–0 Polypropylene suture. Resection of the saccular aneurysm and closure using a small internal thoracic artery patch was then complete. The aortic cross-clamp time was 120 min, and the CPB time was 147 min. The patient had an uneventful hospitalization and was discharged on aspirin therapy. Follow-up multislice CT 10 days after the operation revealed the complete disappearance of the aneurysm and a successful repair with no luminal stenosis by the internal thoracic artery patch. The LITA graft was also found to be patent (Fig. 2). The patient has been followed up yearly since 2009. Fortunately, at the 9-year follow-up, the patient was still asymptomatic, and there were no changes in the ECG and UCG. The patient included in the follow-up had preserved preoperative left ventricular function, and there was no coronary incompetence. Pathology of the aneurysm revealed that the aneurysm wall was very thin due to a lack of trilaminar arterial structure from the remarkable atherosclerotic changes (Fig. 3).

## Discussion & Conclusions

Several operative approaches have been reported for LMCAA, but there is no consensus on whether ligation, repair, or reconstruction is the most appropriate method [2, 3]. LMCA ligation can occlude the important coronary arteries, and there is a possibility that the access route will be lost when future coronary artery stenosis occurs. If a patient needs percutaneous coronary intervention in the future, patch repair of the LMCAA could enable the use of native circulation for access. In previous studies, the saphenous vein patch was the most common choice, and its failure rate was estimated as 5% or less [4]. However, it is not clear whether these productive or degenerative changes result in failure; rather, LMCA atherosclerosis is seen as the culprit. A small internal thoracic artery patch could decrease the incidence of LMCA restenosis and thrombus formation. Therefore, in the present case, we used the distal segment of the LITA as patch material. The rationale is that the internal thoracic artery better resembles coronary arteries than does the saphenous vein in terms of histologic, physiologic, and fibrinolytic properties [5–7]. Use of this small internal thoracic artery patch has the advantages of excellent material strength, similarity to coronary arteries, and availability within the operative field [8].

Coronary angiography remains the gold standard for diagnosis and the best method for the evaluation and assessment of an LMCA aneurysm. Both coronary angiography and intravascular ultrasound offer a good definition of vascular dimensions. However, multislice CT scan, an excellent alternative diagnostic method, is recommended as a postoperative follow-up method for these types of repair, especially for the determination of the exact anatomy and its relation to adjacent structures. Additionally, multislice CT scan is less invasive than coronary angiography and intravascular ultrasound.

This case demonstrates that saccular LMCA aneurysm surgical repair using an internal thoracic artery patch is safe and offers excellent results.

## Abbreviations

CT: Computed tomography
ITA: Internal thoracic artery
LAD: Left anterior descending
LMCA: Left main coronary artery
LMCAA: Left main coronary artery aneurysm
